# Supplementary material for: Comprehensive map and functional annotation of the mouse white adipose tissue proteome
Source: PeerJ. 2019 Jul 25;7:e7352. doi: 10.7717/peerj.7352 (PMC6661141; doi:10.7717/peerj.7352)

Supplemental Fig.S1 Workflow used to profile mouse gonadal WAT proteome.

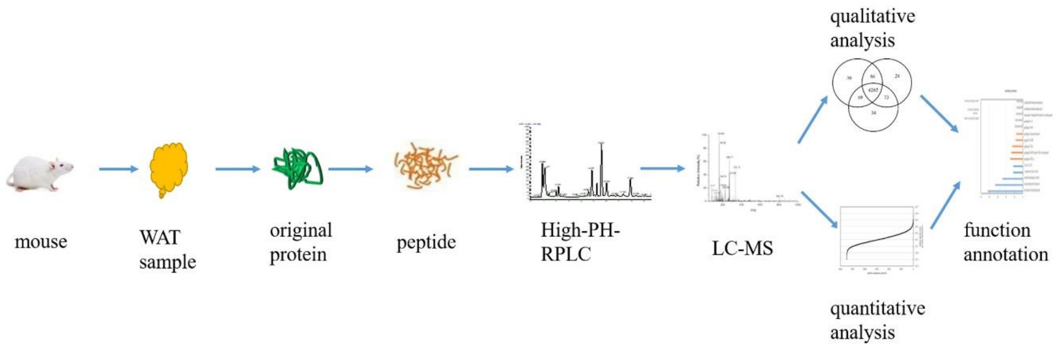

Supplemental Fig.S2 Protein size distribution.

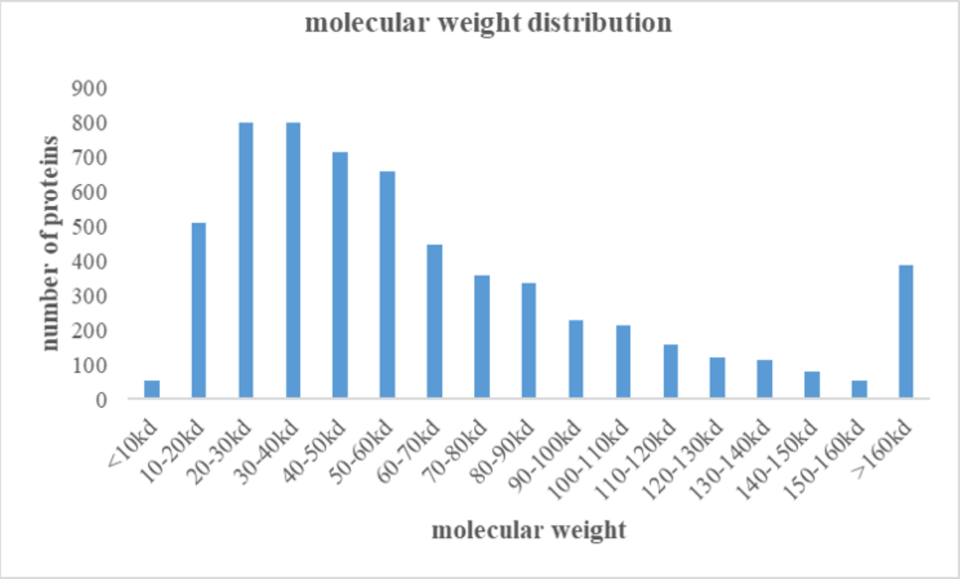

Supplemental Fig.S3 Correlation plot of protein abundance between every two runs (A. Correlation plot of protein abundance between run1 and run2, B. Correlation plot of protein abundance between run1 and run3, C. Correlation plot of protein abundance between run2 and run3).

A

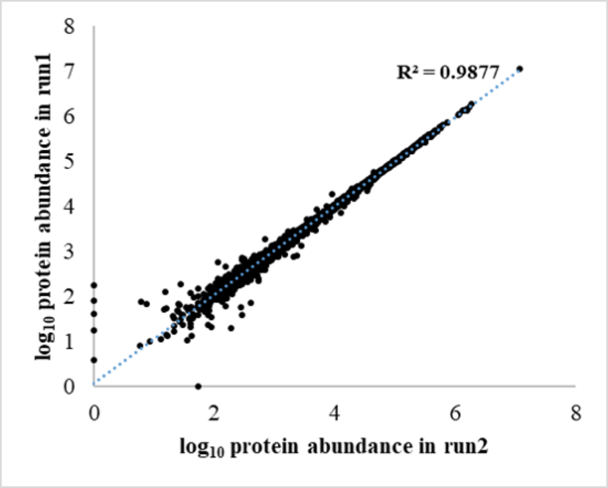

B

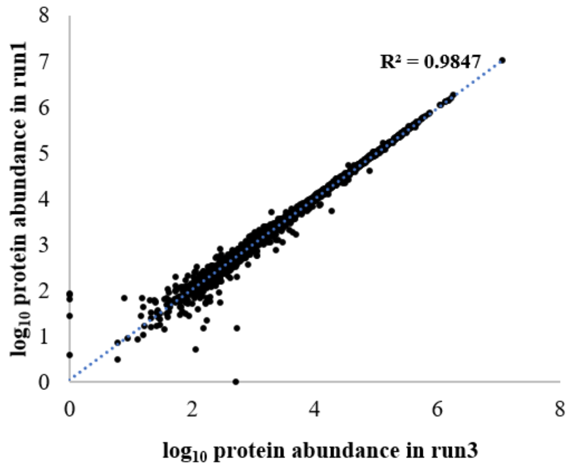

C

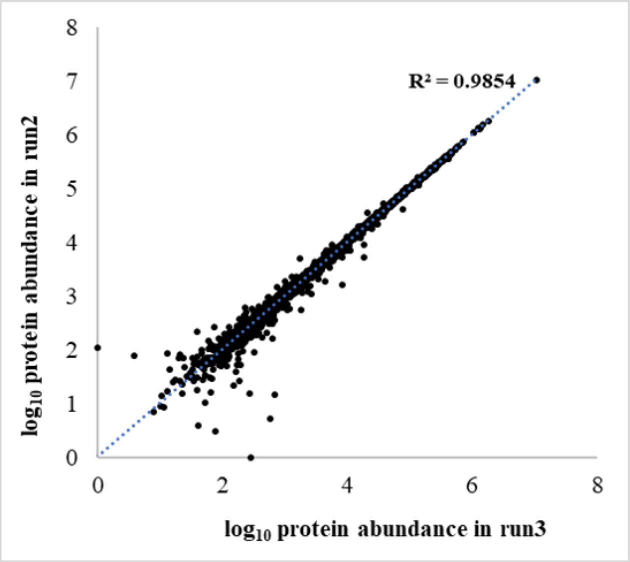

8
7
6
5
4
3
2
1
0
R² = 0.9854
log10 protein abundance in run2
0
2
4
6
8
log10 protein abundance in run3

Supplemental Fig.S4 Venn diagram comparing large-scale studies on the mouse WAT proteome with our high-abundance proteins.

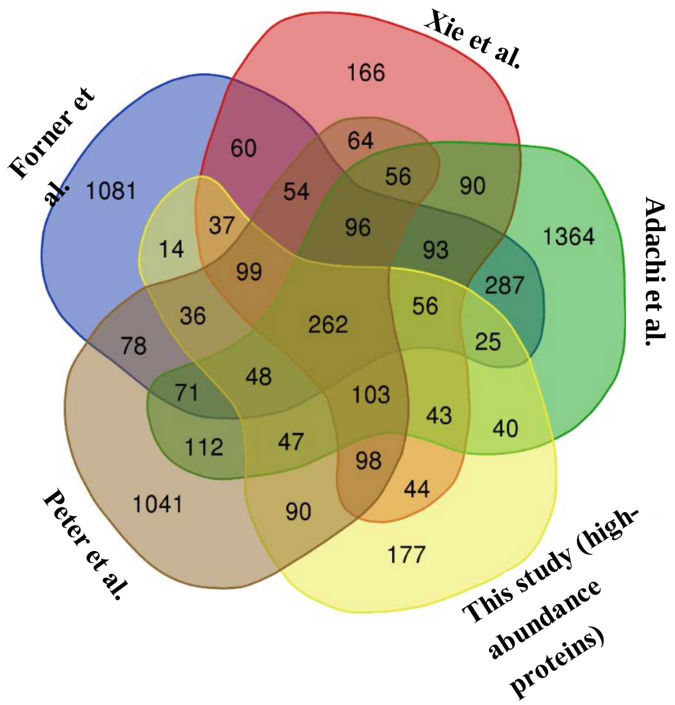

Supplemental Fig.S5 Top canonical pathways in WAT (The horizontal coordinates represent the number of proteins, and the vertical coordinates represent canonical pathways in WAT).

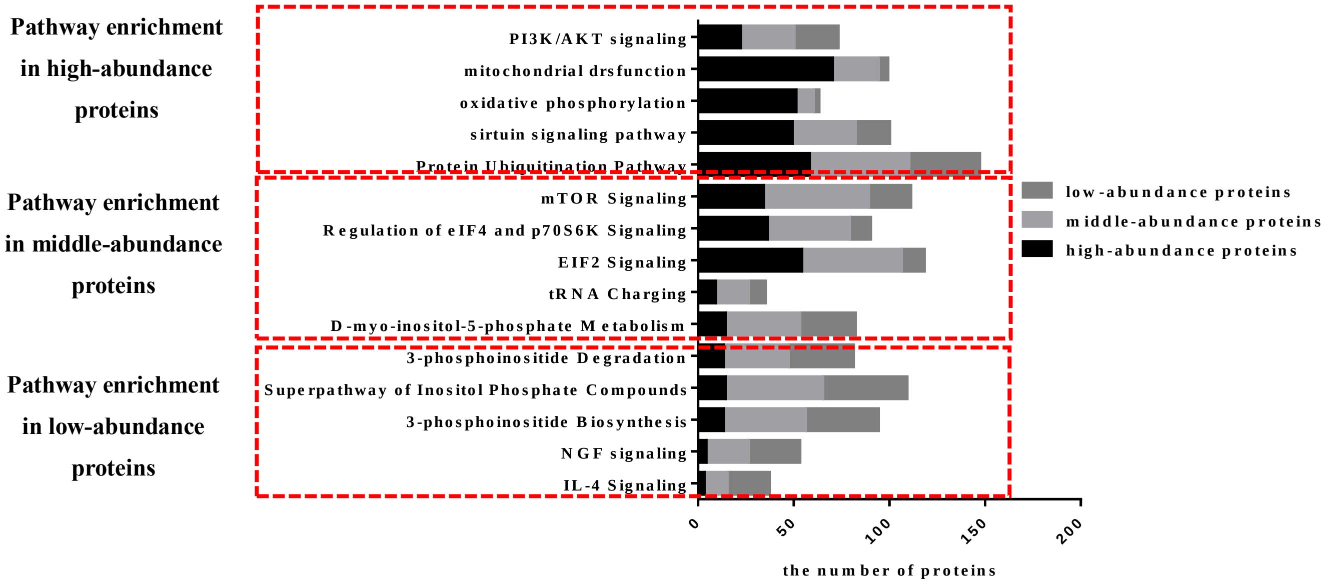

Supplemental Fig.S6 Mitochondrial dysfunction and oxidative phosphorylation signaling pathway (**A**. mitochondrial dysfunction; **B**. oxidative phosphorylation. The red-filled graph represents high-abundance proteins; the gray one is middle-abundance proteins; and the green one is a low-abundance proteins).

A

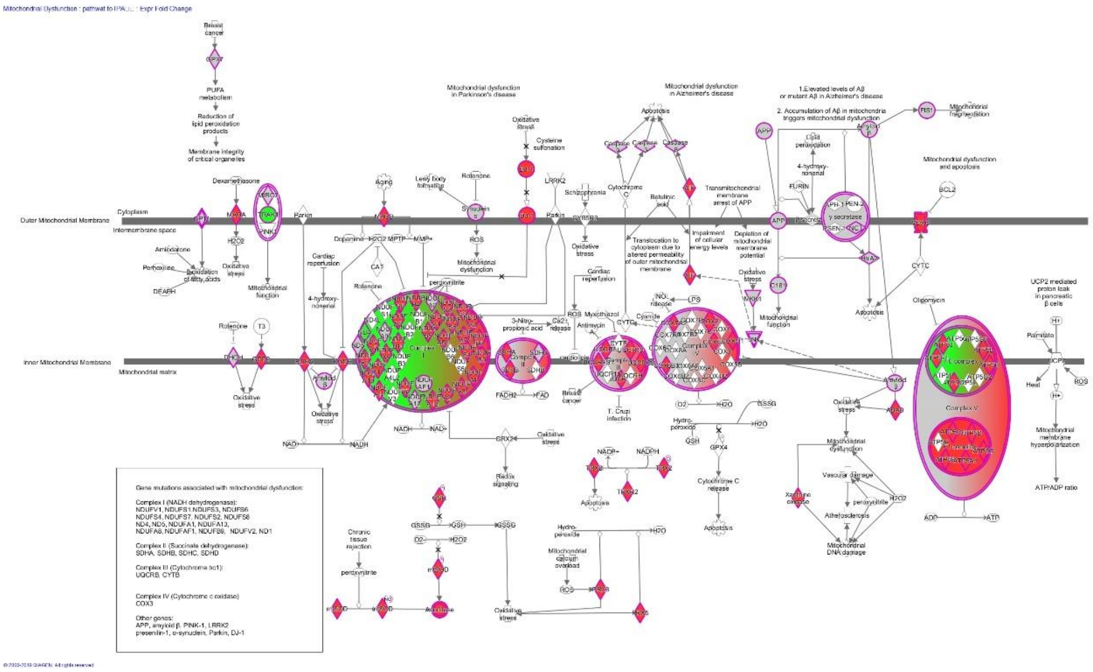

B

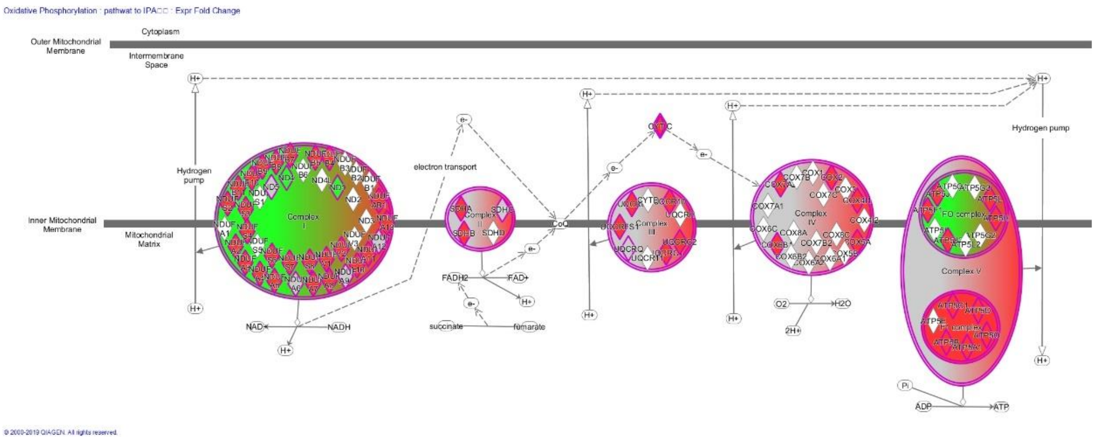

Supplement: Supplemental Information 1 — (A) Correlation plot of protein abundance between run1 and run2. (B) Correlation plot of protein abundance between run1 and run3. (C) Correlation plot of protein abundance between run2. [file peerj-07-7352-s001.pdf]
